# Increased lifespan, decreased mortality, and delayed cognitive decline in osteoarthritis

**DOI:** 10.1038/s41598-019-54867-8

**Published:** 2019-12-09

**Authors:** Anatoly L. Mayburd, Ancha Baranova

**Affiliations:** 1 George Mason University, School of Systems Biology, Manassas, VA 22030 USA; 2 Neurocombinatorix, 5902 Mount Eagle Dr, Suite 1103, Alexandria, VA 22303 USA; 3 Research Centre for Medical Genetics, Moskvorechie str., 1, Moscow, Russia

**Keywords:** Immunosuppression, Alzheimer's disease

## Abstract

In absence of therapies targeting symptomatic dementia, better understanding of the biology underlying a cognitive decline is warranted. Here we present the results of a meta-analysis of the impact of osteoarthritis (OA) on cognitive decline and overall mortality. Across 7 independent datasets obtained in studies of populations in the USA, EU and Australia (NBER, NSHAP, TILDA, NACC, Kaiser Permanente, GRIM BOOKS, OAI, with a total of >7 × 10^7^ profiles), OA cohorts demonstrated higher cognitive scores, later dementia onset as well as longer lifespan and lower age-specific all-cause mortality. Moreover, generalized OA with multiple localizations is associated with more significant reduction of mortality and dementia than a singly localized OA or no arthritis. In OA patients with younger ages, all-cause mortality was disproportionally reduced as compared to that in controls, while exponential term of Gompert’z hazard function was increased, accelerating mortality accrual at later ages. Up to 8–10% of poly-osteoarthritic patients are predicted and observed to reach centenarian lifespan, while in matched non-OA population the same benchmark is reached by less than 1% of patients. These results point at a possibility of life-extending and cognition preserving impacts of OA-conditioned immune system.

## Introduction

Dementia is a major condition to impact aging populations; by 2050, up to 131 million individuals globally are projected to suffer from its subtypes^1^. So far, no disease-modifying therapies produced significant reversal of a clinically evident cognitive decline^2^. Respectively, preventive approaches addressing the earliest stages of cognitive decline, which are likely to be more amenable to intervention^3^, are warranted.

One area of intense interest is re-purposing of already approved pharmaceuticals or over-the-counter supplements as possible components of a combination therapy at once addressing many pathophysiological components of dementia^4,5^. It is tempting to speculate that potentially protective therapeutic or even preventive combinations may be teased out of the data describing life trajectories of multi-morbid patients exposed to polypharmacy prescribed for various health concerns, especially for patients of advanced age^4,5^. In this light, analyses of population databases and published clinical study datasets may hold a substantial promise^6^.

In addition to potential effects of disease-associated polypharmacy, other prevalent factors, such as the presence of various comorbidities, may interfere with dementia-promoting pathophysiological processes. For example, while the levels of uric acid are recognized as an independent cardiovascular risk factor^7,8^, a reduction for dementia risk is notable in patients with either gout or asymptomatic hyperuricemia^9–11^, and is independent of either presence or absence of anti-hyperuricemic treatment^11^.

Osteoarthritis (OA) impacts 20–30% of elderly population, with the higher prevalence in females^12^. Established diagnosis of OA is associated with substantial pain and related immobility^13^. The modern view of osteoarthritis defines it as a systemic low-grade inflammation state^14^, accompanied by aberrant immune-metabolic signature^15^ and dysregulated autophagy^16^. It is of interest that multiple research groups independently reported decreased mortality rates in osteoarthritis^17–20^. Observed decreases in all-cause mortality were specific for osteoarthritis (OA), while for a somewhat related disease – rheumatoid arthritis - respective rates were significantly increased^21^. Notably, the comorbidity rates were elevated in both conditions comparably^22^. Thus, some non-comorbidity related factor is likely to be at play, and is prominent in differentiation of OA and RA cohorts by the mortality rates.

As peripheral chronic inflammatory states were shown to correlate with cognitive status^23^, we set to explore the effects of osteoarthritis on a rate of cognitive decline. The current publication field focusing on OA and dementia specifically is scant and inconsistent, with a general trend pointing that OA diagnosis is a detriment to cognitive health^24–28^. Out of 11 independent sources considered in^24–28^, 8 mention increased risk of dementia in osteoarthritis, while 3 report a decreased risk. Previous studies showed that late-life cognitive changes are related to functional health and proximity to the end of life^29^. Therefore, profile of age-specific mortality in OA may influence the relative risk of dementia. Here we analyze cognitive decline and mortality in OA as two connected events. Our motivation was to reproducibly identify population subsets with significantly increased longevity (>10 years) and simultaneously decreased dementia, thus enabling further differential studies involving general population controls, and subsequent dissection of particular pathophysiological mediators of delayed ageing and cognitive decline.

## Results

### Osteoarthritis is associated with delayed cognitive decline, decreased mortality and altered profile of adverse event accumulation

**Figure 1:**
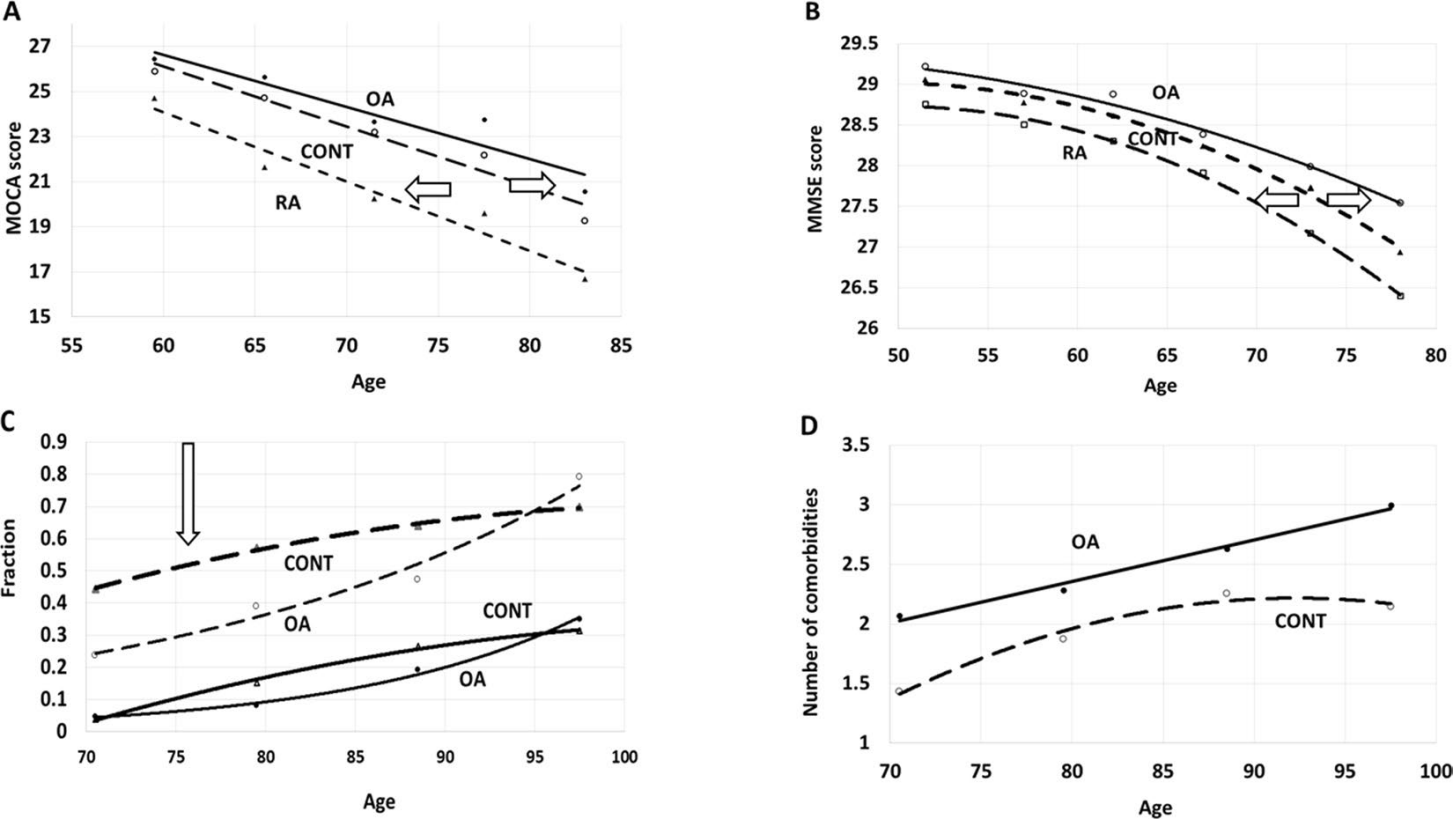
Qualitative trend introduction. Dependence of cognitive status, mortality rate and comorbidity accumulation as a function of age in osteoarthritic (OA), non-arthritis control (CONT) and rheumatoid arthritis (RA) patients. In all cohorts the patients were age- and gender- matched. Other confounders were measured and controlled in the cohorts (Table 1). (**A**) Dependence of Montreal Cognitive Score (MOCA) on age in OA (black circles, N = 350 patients), CONT (empty circles, N = 1250) and RA cohorts (triangles), N = 250), measured in NSHAP dataset. Arrows symbolize differences between the ages when the measured parameter is equal in the compared cohorts. (**B**) Dependence of Multiple Mini-State Score (MMSE) on age in OA (white circles, N = 1050), CONT (black triangles, N = 4800), RA (white squares, N = 710), measured in TILDA WAVE1 dataset. Arrows symbolize differences between the ages when the measured parameter is equal in the compared cohorts. MMSE scores were the averages between the values available for the patients in the WAVES 1 to 3. (**C**) Dependence of all-cause mortality (two upper curves) and all-cause dementia (two lower curves) on the patient’s age in Kaiser Permanente study of the oldest old, 1971–1988. The order of the curves, from top to bottom in the direction of the arrow: mortality in non-arthritic control (N = 2900), mortality in osteoarthritis (N = 1050), dementia in non-arthritic control (N = 2900), dementia in osteoarthritis (N = 1050). (**D**) Age dependence of the average number of comorbidities measured in a patient in Kaiser Permanente study of the oldest old, 1971–1988. The patients were observed in OA (N = 1050) and CONT (N = 2900) cohorts. The comorbidity panel included cardiovascular, pulmonary, renal, oncological diseases, diabetes, endocrine problems, dementias and depression. Immunological diseases (dermatitis, asthma) were included, however, arthritis was excluded in order to stress upon non-arthritis component of comorbidity.

Figure 1 presents cognitive performance data collected in osteoarthritis (OA), non-arthritic control (CONT) and rheumatoid arthritis (RA) cohorts in three independent datasets, NSHAP^30^, TILDA^31^, and Kaiser Permanente^32^. Figure 1A,B show that initial cognitive scores are higher in OA patients as compared to the control and to the RA cohorts. Rheumatoid arthritis (RA) cohort was included in the study as a positive control due to well-established increase in mortality observed in RA as compared to non-arthritis cohorts. If our methodology is correct, we expect to re-capture this effect in datasets analyzed. After assessment with either MOCA or MMSE questionnaires, in patients with OA cognitive decline was delayed, on average, by 4 year as compared to non-arthritic control, and by 8–16 years than in a cohort with RA.

Figure 1C shows an OA vs control cohort comparison of all-cause dementia and all-cause mortality accumulation as a function of age. The dataset was normalized by age and gender only, while comorbidity levels were as observed in each of the respective cohorts. Figure 1D demonstrates that in the entire range of ages, patients with OA accumulated the comorbidities at substantially higher rates (p = 3 × 10^−41^) than in non-arthritic cohort. Notably, in OA patients, higher comorbidity loads had not translated into respective increases in the incidences of all-cause mortality and dementia, which remained lower than in controls up to the age of 90 and older. Furthermore, the profiles of cognitive decline in patients with OA and in non-arthritic controls were different. When the cohorts were divided into quartiles by age, both the dementia rates and mortality rates in younger OA patients were lower, following by increases in later life. By contrast, in non-arthritis controls, age-dependent profiles of mortality and dementia were more linear since inception, resembling that of 8–12 years older patients with OA.

**Figure 2:**
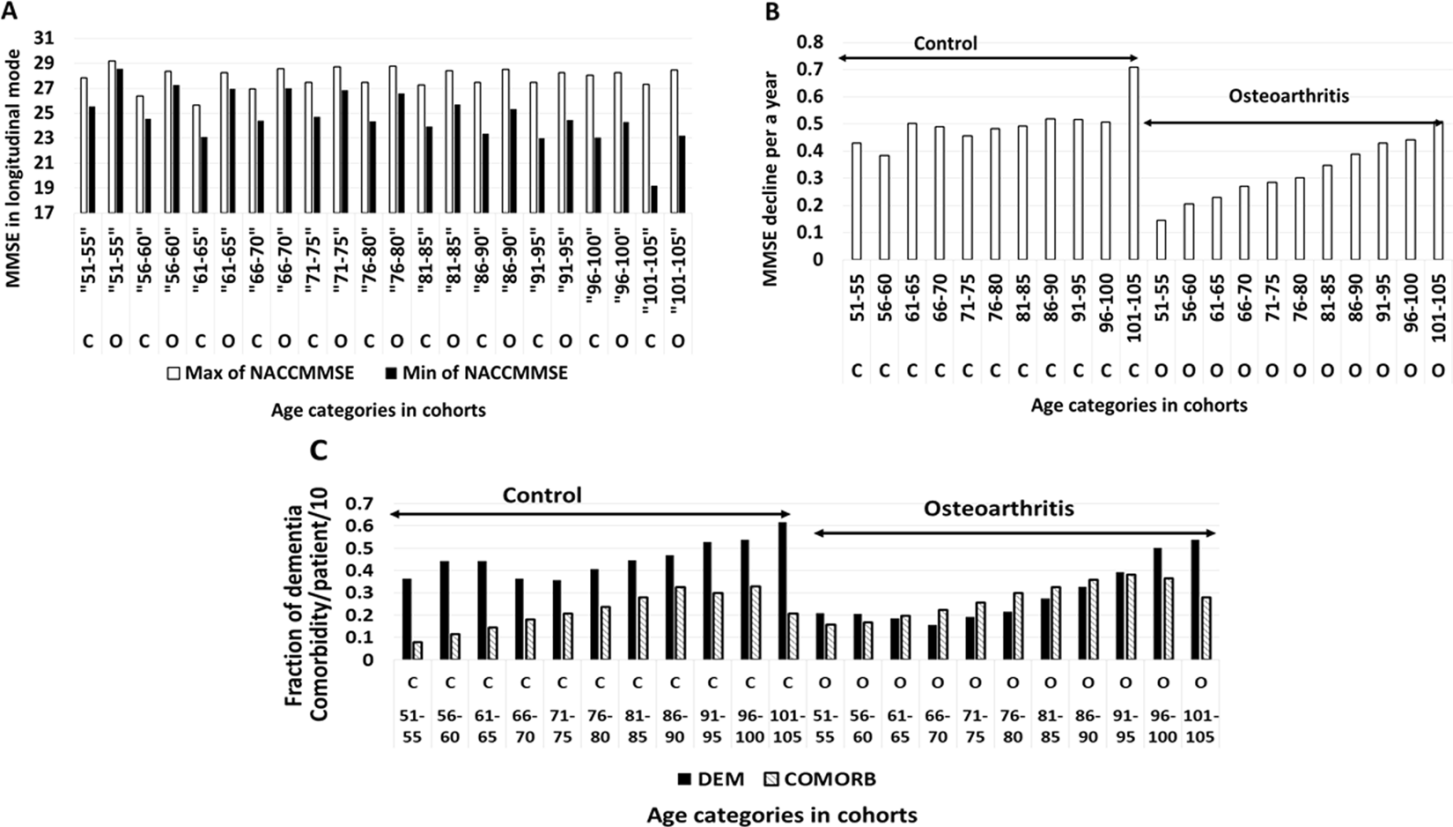
Qualitative comparison of absolute MMSE score values (**A**), patient-level averaged MMSE declines (**B**), dementia fractions and comorbidity rates per a patient (**C**) in OA (N = 8525) and control (N = 5250) cohorts of NACC, gender-matched. (**A**) In each age sub-range, MMSE was measured at the beginning and at the end of follow up, in both control (C) and osteoarthritis cohorts (O). (**B**) In each age sub-range, MMSE declines were measured individually in each patient and normalized to the individual follow up lengths, in both control (C) and osteoarthritis cohorts (O). (**C**) In each age sub-range, fraction of dementia (black bars) and number of comorbidities per a patient (brought to the range) measured in both control (C) and osteoarthritis cohorts (O).

Cohort-specific trends revealed by analysis of NSHAP, TILDA and Kaiser Permanente datasets were independently validated by exploring NACC (National Alzheimer’s Coordination Center^33^) database (Fig. 2). In gender-matched OA and control cohorts from NACC, average MMSE scores were computed for identical age intervals (Fig. 2A). Figure 2B shows the patient-specific differences between the initial MMSE and final MMSE score normalized to the length of follow up and further summarized for each age interval. The plotting shows that, in patients with OA, average MMSE scores were higher than that for non-arthritic controls for 10 out of 11 age intervals, while the normalized differences were higher for each of 11 age intervals. In the NACC, the trends in MMSE declines, as well as an accrual of dementia and comorbidities (Fig. 2B,C) resembled the trends uncovered by analysis of Kaiser Permanente database (Fig. 1C,D), pointing at relatively greater protection detected in younger OA cohorts.

For more detailed analysis aimed at discerning specific cognitive components selectively modified by the presence of OA, we studied the TILDA and NACC datasets as the most elaborate and recent, 2009–2015 and 2005–2018, respectively (Table 1). In this analysis, the following parameters were controlled: age, gender, health status, education and income level. Of several types of cognitive deficiencies tested in comparison of OA and non-arthritis cohorts, only short-term and long-term recalls were found differing by T-test; both were less impaired in OA cohort profiled in TILDA. In addition, fraction of patients with low values for the integral MOCA and MMSE scores in OA cohort was lower, which together with values of recall contributed to the summary metric of cognitive risk, also significantly different between patients with OA and non-arthritic controls.

**Table 1:** Quantitative comparison of specific components of cognitive performance between osteoarthritis cohort in TILDA WAVE1 (or NACC) and age/gender matched controls.

Controlled parameter (confounder)	OA	CONT	Compared parameter	OA	CONT	p-value
Males (TILDA)	0.26	0.28	Attrition (TILDA)	0.15	0.18	0.024
Age (TILDA)	69.9	69.9	MMSE average (TILDA)	28.1	27.8	0.009
Income (TILDA)	2.97	2.93	MMSE average max (NACC)	28.55	27.12	3.8 × 10^−62^
Education (NACC)	15.8	16.3	MMSE average min (NACC)	26.12	24.12	7.8 × 10^−60^
Education (TILDA)	3.59	3.48	Cognitive Risk (TILDA)	0.38	0.51	0.002
Stroke (TILDA)	0.018	0.019	Poor short-term recall (TILDA)	0.137	0.192	0.005
Somatic Risk (TILDA)	0.36	0.33	Poor long-term recall (TILDA)	0.07	0.101	0.057
Age at baseline (NACC)	77.2	71.8	Educational underperformance (NACC)	0.076	0.057	0.066
Males (NACC)	0.37	0.37	Advanced degrees (NACC)	2.64	2.82	0.1
Stroke (NACC)	0.043	0.029	Dementia in the baseline (NACC)	0.14	0.29	3.8 × 10^−109^
Somatic risk (NACC)	2.85	2.02	Dementia in the end of follow up (NACC)	0.24	0.40	4.7 × 10^−95^

In TILDA, osteoarthritis cohort (OA, N = 820) and non-arthritic controls (CONT, N = 2300) were normalized by age, gender and somatic disease risk. Somatic comorbidity panel (Somatic Risk) included open-heart surgery, heart attack, angioplasty and stent installation, congestive heart failure, cardio-pulmonary deficiency that requires oxygen supply, cancer, stroke and diabetes. Income levels were provided as a strata of population rank.

The compared parameters in TILDA include: Attrition – fraction of patients not re-appearing in WAVE2 of TILDA after being present in WAVE1, MMSE average – is average score between WAVE1-WAVE3, Educational under-performance – completion of only elementary school, Cognitive risk – the sum of fractions for low readings of MOCA (<20), MMSE (<20), poor short and long term recalls, poor visual recognition and reasoning, high delays in performing test assignments. Recall tests were conducted according to MOCA methodology.

The compared parameters in NACC Version 3 (OA, N = 8525, CONT, N = 5250) included dementia fraction, MMSE at the beginning and the end of follow up (MMSE MAX and MMSE MIN), educational underperformance (average years of underperformance per a patient) and advanced degree presence (average years of advanced education per a patient). Educational underperformance was defined in NACC as <9 years of schooling, advanced degree as >18 years of schooling, stroke and dementia rates in NACC are provided as fractions, somatic risk as number of comorbidities per a patient in the cohort.

Analysis of substantially larger NACC dataset confirmed these trends (Table 1), especially that of higher cognitive baseline and slower rate of decline observed in a cohort with OA. These trends are even more striking when cohort-specific distributions of age were accounted for. These observations confirm the presence of the cognitive aging gap between OA and non-arthritis cohorts presented at Fig. 1. It was previously suggested that in some cohort comparisons cognitive aging gaps may be explained by initially unequal cognitive reserves secondary to educational disparity^34^. In present analysis, however, amounts of educational underperformers were higher, and average years of advanced education in OA cohort were marginally lower than that in non-arthritis controls, thus, excluding possibility of educational disparity bias. In summary, analysis of NACC database shows that patients with OA had lesser propensity to progress to dementia, while, on average, being older, carrying higher comorbidity burden, and attaining lower education levels than that in non-arthritic controls.

### Decreased mortality in OA is confirmed by meta-analysis of additional datasets and of published literature

**Figure 3:**
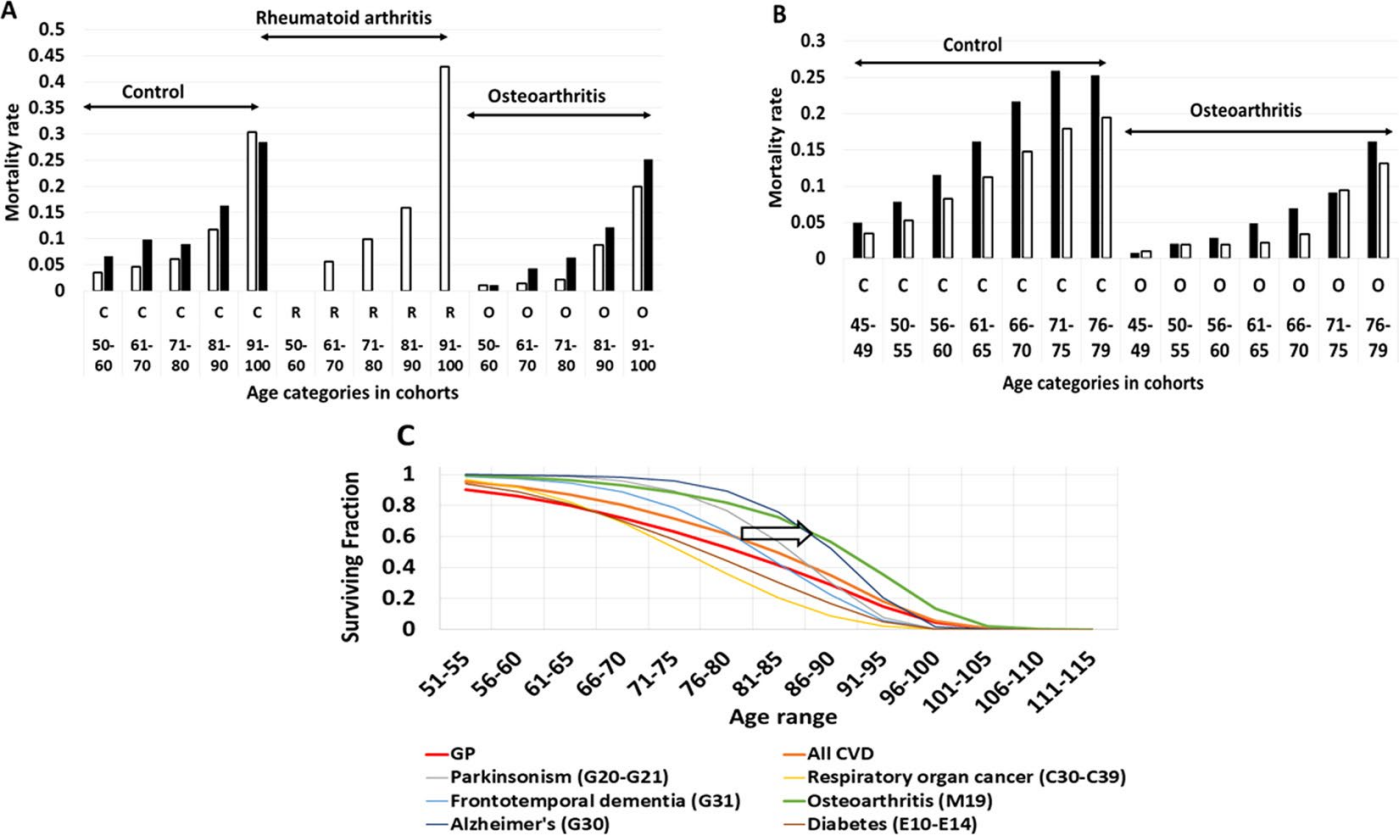
Qualitative trend review. Mortality rates and survival in osteoarthritis cohorts of NACC, OAI and NBER datasets as a function of age. (**A**) National Alzheimer Coordination Center (NACC) dataset includes 8300 patients in osteoarthritis cohort, 300 in rheumatoid arthritis and 7000 in non-arthritis control. The age ranges with insufficient population to ensure decreased random error were discarded (<50 and >100). Only clinician-selected diagnoses (variable ARTYPE) of Version 3.0 were accepted, excluding self-reporting data. Males and females were considered separately (white bars indicate data for females, black bars for males). Due to small cohort size, data for rheumatoid arthritis are available only in females. (**B**) Osteoarthritis Initiative (OAI) dataset includes 4800 patients in osteoarthritis cohort. The control was provided by computing the risk of dying using actuarial tables for the years when the project was active, considering the initial ages, years in follow up and withdrawals. The probability of survival at the beginning and at the end of observation was compared by integral Gompertz model (4) in the control. Males and females were considered separately (see legend for Fig. 3A). (**C**) Fractions of survival in the NBER populations marked by the tags of interest (2 × 10^4^ osteoarthritis of hand, M19* ICD-10 code, 4 × 10^6^ in general population control). Red line symbolizes survival in general population (GP), orange line symbolizes survival in any of the sum of cardiovascular disease (infarction, congestive heart failure, angina, arrythmia, fibrillation, stent, aneurisms, valvular deformations, endocarditis, myocarditis, pericarditis, atherosclerotic cardiac disease). The CVD survival (orange line) is shifted to the right vs. GP due to CVD development mostly in later life, thus starting extinction later. Osteoarthritis is represented by the green line and it emerges concurrently with CVD. Diabetes, lung cancer and early dementia are known as the strongest predictors of shortened lifespan and thus serve as positive controls, producing left-shifts vs. general population and CVD curve. Osteoarthritis produces a comparable right shift on the same plot, indicated by the arrow.

Figure 3 combines measurements of mortality and survival in three additional datasets providing arthritis-related labels. Figure 3A compares age-distribution of all-cause mortality in age and gender-matched controls (N = 7000), as well as in patients with rheumatoid arthritis (N = 300) and osteoarthritis (N = 8300) profiled by National Alzheimer Coordination Center (NACC). In all Intervals of age, the mortality rates follow the order: osteoarthritis < controls < rheumatoid arthritis. In NACC analysis, protective effects of OA were detectable for both genders. Effect magnitudes were greater in relatively younger age intervals, while diminishing at age intervals of 80 and older. Analysis of the data collected by Osteoarthritis Initiative (OAI) presents similar trends^35^. In lieu of mortality data collected in a matched non-arthritis cohort, which was too small in OAI dataset (N = 100 patients), the mortality risks were computed using actuarial tables, considering the ages of the patients and patient-specific duration of the follow up. External control was employed to compare the mortality risks with observed mortality (Fig. 3B).

Decreased mortality rates are reflected by improved survival at older ages and longer lifespans. For NBER populations tagged with ICD-10 code M19* (osteoarthritis of hand) and/or J* code (cardiovascular diseases, all), the survivals are traced at Fig. 3C. For population with OA, survival curves (green line) are shifted to the right by 10–12 years when compared to populations with CVD or when compared to populations with rheumatoid arthritis (ICD-10 codes M05-06*, not shown). Notably, cardiovascular diseases (CVD) emerge synchronously with osteoarthritis as a function of age (data not shown). For comparison purposes, the survivals for cohorts with cancer of respiratory organs, diabetes and dementias were also plotted alongside OA and CVD. These diseases were selected as benchmarks as known causes of premature mortality with age-dependent accumulations of initial diagnosis similarly to OA or CVD. With CVD, OA and diabetes populations emerging in the same age-specific brackets, the difference in relative survival is drastic as measured in 4 × 10^6^ profiles of NBER.

The results produced in our meta-analysis of data sources agree with the meta-analysis of published literature (Table 2).

**Table 2:** Meta-analysis of published literature, addressing mortality trends in osteoarthritis patients.

#	PMID	N	HR	#	PMID	N	HR
Meta-analysis of published literature							
1	30169829[d]	5.2 × 10^4^	1.3/1, K-RKOA	18	20416065	2163	0.92 HRE
2	29588472*[sec]	809	0.76/1.1 HRE/KRE	19	17540743	6.4 × 10^4^	0.77 HRE, KRE < 12 years follow up
3	28754502	601	1.97 K	20	12784389	2.35 × 10^6^	1.0 OA
4	28607349[w]	1025	1.98 K	21	12525385	7200	1.1 H
5	27866950	4278	0.86 KRE/HRE	22	10743987	3.9 × 10^4^	0.73 HRE
6	26543059	821 (K), 806(H)	2.0, 1.0 K/H	23	28375889*	7.1 × 10^5^	0.6 HRE/KRE
7	26986874[d]	5.2 × 10^5^	0.9 K, HP	24	29489471*	1.3 × 10^5^	0.98 HRE
8	17462546[ed]	7.4 × 10^5^	0.72	25	26704362	5853	0.9 SAR
9	26777550*	3625	0.81 KRE/HRE	26	29158649	358	0.91 KRE
10	26751980*	9443	0.73 (K, <10 years follow up), 1.9 (>20 years follow up)	27	26871792*	3.6 × 10^5^	0.84 (at 5 years follow up), 1.1 (>10 years follow up)
11	24047870	1348	1.0 H	28	28754502	601	1.97 K
12	25778744	7889	1.14 HP	29	25179456	850	0.5 OA
13	25819581	1025	1.6 K	30	21385807	1163	1.55 OA
14	24608134	2125	1.27 K	31	21282754	1.7 × 10^5^	0.7 HRE
15	15252092	8000	1.32 H	32	24158755	1290	1.4 OA
16	doi: 10.1093/rheumatology/kez108.062	8066	1.18 H, K	33	30583096	4182	1.15 K
17	30559011*	4907	0.95 HRE 1.45 HRE	34	30302017*	1.3 × 10^6^	0.65 (3 years) 0.85 (7 years), HRE, KRE

Meta-analysis of literature includes PubMed ID number of the source (PMID), the size of the study (N), the average hazard ratio in sub-cohorts (HR) for all-cause mortality in OA, K – knee generic, RKOA – knee radiographic, KSR – knee self-reported, H - hand generic, HP – hip generic, HRE – hip replacement, KRE- knee replacement, OA – osteoarthritis generic, SAR – shoulder arthroplastic, SEC – secondary, P – primary.

*The publications describing initial decrease of all-cause mortality followed by subsequent increase; [w] The publications describing the effect of decreased mobility associated with knee OA on all-cause mortality, [ed] Mutual exclusion of diseases, [d] Reductions in selected co-morbidities vs. control.

For meta-analysis, the individual hazard ratios were integrated based on numbers of patients in each study, with a total of 6.4 × 10^6^ patients analyzed in 34 source that passed the inclusion criteria (Methods).

### Supplemental meta-analysis file is here

The hazard ratios of all-cause mortality in each independent source could be presented as either one value or a range of values with the lower and upper boundaries. The lower boundary hazard ratios typically belong to the code describing OA of hand and to shorter follow-ups. The upper boundary ratios belong to the code describing knee OA and to longer follow-ups. The individual lower and upper boundary hazard ratios were processed separately, forming the boundaries for the entire meta-analysis. For the lower boundary, 3.94 × 10^6^ patients were in the 18 cohorts with HR < 1.0, 4.3 × 10^4^, in the 12 cohorts with HR >1.0, and 2.5 × 10^6^ in the 4 neutral cohorts with the HR = 1.0 (unchanged vs. control). The meta-analysis weighted average for a hazard ratio was 0.82. For the upper boundary, 3.5 × 10^6^ patients were in the 12 cohorts with HR < 1.0, 4.7 × 10^5^ in the 17 cohorts with HR >1.0, and 2.5 × 10^6^ in the 5 neutral cohorts with the HR = 1.0. The meta-analysis weighted average was 0.88 on the upper boundary. These results indicate that decreased mortality is statistically more dominant across various data sources, OA definitions in the datasets, cohort-specific biases and methods of normalization. The odds ratio of a random patient with osteoarthritis tag to reach a lifespan longer than in control was at ~ 1.6 on the lower boundary and at ~ 1.4 on the upper boundary, based on the ratios of populations in the cohorts with decreased vs. unchanged ratio of all-cause mortality. The sources #8, 9, 19, 22, 23, 29, 31 and 34 demonstrate HR in the range 0.5–0.81, comparable with the quantitative ranges shown in this report (Figs. 1–3) at least in some age strata, incorporating 3.02 × 10^6^ patients in these cohorts. The contrary sources with HR >1.2 (#1, 3, 6, 10, 13–15, 17, 28, 30, 32) include 8.2 × 10^4^ patients at the same distance from HR = 1.0 as the group with decreased HR. It is apparent that statistical distribution of patients in all pooled cohorts of meta-analysis is shifted in favor of HR decrease. Some literature sources, which are marked by an asterisk, contain datasets best described as two-stage time-course of all-cause mortality, when initial decreases in mortality are followed by its increase at longer follow-ups. These references agree with the trends of Figs. 1–3, also showing relative decrease of OA mortality in younger cohorts followed by accelerated mortality accrual later in life.

### Gompertz model constants are altered in osteoarthritis

The decrease of mortality seen in younger OA cohorts may be expressed quantitatively by Gompertz constants, which allow estimation of the population fraction that exceeds centenarian lifespan (Table 3).

**Table 3:** Determining a percent of population exceeding 100-year benchmark in different cohorts, using extraction of Gompertz constants from AMRT metrics and equivalents.

COHORT	AGES												
	48	53	58		63	68	73	78	83		88	93	98
AT	0.0042	0.0065	0.01		0.0156	0.024	0.038	0.0585	0.0909		0.1413	0.2194	0.3409
NACC CN*	0.010	0.015	0.022		0.031	0.044	0.064	0.090	0.129		0.185	0.264	0.38
NACC OA*	0.003	0.005	0.008		0.126	0.20	0.031	0.050	0.079		0.125	0.198	0.313
OAI* OA	0.0017	0.0026	0.0041		0.0064	0.01	0.0156	0.0245	0.0385		0.0603	0.0945	0.148
KAISER OA	0.002	0.003	0.0049		0.0078	0.012	0.019	0.0304	0.048		0.0755	0.119	0.187
KAISER NON-OA	0.007	0.01	0.015		0.0215	0.031	0.045	0.065	0.095		0.137	0.199	0.289
TILDA* OA	0.0024	0.0042	0.0069		0.0115	0.019	0.031	0.052	0.088		0.147	0.246	0.416
NBERC	0.0033	0.0052	0.00825		0.01315	0.021	0.0335	0.0533	0.0852		0.1358	0.2167	0.347
NBER-M19 OA	0.0009	0.0016	0.0029		0.0053	0.0095	0.017	0.0315	0.0572		0.1043	0.1896	0.345
NBER - M05-M06 RA	0.006	0.0095	0.0144		0.0218	0.033	0.05	0.0761	0.1153		0.1749	0.2653	0.402
NBER- M17 OA	0.0036	0.0056	0.0087		0.0134	0.021	0.032	0.0498	0.077		0.1193	0.1846	0.286
NBER -M15 OA	0.0017	0.003	0.0048		0.0079	0.013	0.022	0.0364	0.0606		0.1008	0.1677	0.279
GRIM BOOKS GP	0.0027	0.0044	0.0069		0.011	0.0174	0.0276	0.0439	0.0697		0.1106	0.1755	0.2786
GRIM BOOKS OA	0.0009	0.0015	0.0025		0.0043	0.0074	0.0126	0.0215	0.0367		0.0628	0.1072	0.1832
GRIM BOOKS RA	0.0024	0.0039	0.0065		0.0108	0.0178	0.0294	0.0486	0.0802		0.1325	0.2189	0.3617
**Gompertz constants, ln H(T) = ln [a] + b × T**													
**COHORT**	**Ln [a]**			**[B]**			**Regression residual**			**Predicted percent reaching 100 year lifespan**			
AT	−9.71 ± (0.69)			0.0881 ± (0.0063)			0.0102			1.1%			
NACC CN*	−7.97 ± (0.42)			0.0714 ± (0.0038)			0.0056			0.2%			
NACC OA*	−10.167 ± (0.78)			0.0919 ± (0.007)			0.012			1.6%			
OAI*	−10.72 ± (0.54)			0.0899 ± (0.0045)			0.0051			13%			
KAISER NON-OA*	−8.52 ± (0.28)			0.0743 ± (0.0025)			0.0022			1%			
KAISER OA*	−10.57 ± (0.58)			0.0908 ± (0.0049)			0.006			8.4%			
TILDA OA*	−10–12* (estimated)			0.0935–0.1141 (estimated)			NA			0.6–0.8%			
NBERC	−10.37 ± (0.127)			0.0954 ± (0.0012)			0.0003			1.06%			
NBEROA-M19	−12.95 ± (0.24)			0.1217 ± (0.0023)			0.0007			3.3%			
NBER - M05-M06	−9.21 ± (0.61)			0.0833 ± (0.0051)			0.0074			0.7%			
NBER- M17	−9.81 ± (0.59)			0.0874 ± (0.0052)			0.0072			2.0%			
NBER -M15	−11.25 ± (0.25)			0.1018 ± (0.0022)			0.0010			8.4%			
GRIM BOOKS RA	−10.85 ± (0.98)			0.1004 ± (0.0090)			0.0162			1.2%			
GRIM BOOKS OA	−12.19 ± (0.64)			0.1071 ± (0.0056)			0.0055			12%			
GRIM BOOKS GP	−10.33 ± (0.42)			0.0924 ± (0.0037)			0.0033			2.6%			

The leading section represents H(T) – distribution of mortality hazard as a function of age.

Control cohorts include actuarial tables, average for 2005 and 2015 (AT, see https://www.ssa.gov/oact/STATS/table4c6.html), NBERC – general population control derived in NBER, NBER RA - rheumatoid arthritis control in NBER, NACC CN – non-arthritis control NACC population, KAISER non-OA – Kaiser Permanente study non-arthritic population, GRIM BOOKS GP – general population in GRIM BOOKS, GRIM BOOKS RA – rheumatoid arthritis control.

OA cohorts include NACC OA cohorts, OAI - OAI cohorts, M15* – patients with poly-arthrosis (ICD-10 code M15), NBEROA-M19* cohorts (osteoarthritis hand localization), NBEROA-M17* (osteoarthritis knee localization), KAISER OA (all localizations), TILDA* (mortality for this dataset was estimated from cognitive decline risk).

The symbol [*] indicates the datasets where constants were extracted in the mixed living-decedent populations with a limited follow up. The rest (AT, GRIM BOOKS and NBER data) present constants extracted in decedent data only with a follow up equal to a lifespan.

[a,b] Are pre-exponential and exponential Gompertz constants respectively.

The analysis of Table 3 was started with actuarial data processed as described by the Methods. In this cohort, values of Gompertz constant agree with literature^36,37^. Comparative analysis of cohorts with OA and matched non-arthritis controls indicates 3-fold lower probability of dying in the age range of 48–60 years, 2-fold lower probability at 60–80 years, and 1.5-fold in 80–100 years (cohorts: NACC, KAISER, OAI, GRIM BOOKS). These differences translate into 6 to 8-fold lower probability of achieving 100 years old benchmark lifespan for non-OA control individuals in all compared cohort pairs. In NACC, the prevalence of dementia is higher than in other databases, which contributes to NACC-specific shorter lifespans. Analysis of NBER cohorts confirms this observation directly, by showing that approximately 3.3% fraction of M19* ICD-10 code carriers (hand and shoulder OA) reach an age of 100 years in NBER, while in general USA population only 1.1% reaches the same benchmark (Fig. 3C), with the prevalence of OA diagnosis in centenarians being at 30%.

The MCD data from Australia (GRIM BOOKS) indicate that 12.5% of M19* ICD-10 code carriers have lifespans longer than 100 years. Life expectancy is higher in Australia (82.5 years vs. 78 in the USA), and the percentage of non-arthritis control individuals reaching the same benchmark is at 2.5%. Across three different Western societies, namely Ireland, USA and Australia, profiled in the report, the ratios of the surviving percentages for OA and controls were similar in all comparable cohort pairings, reaching odds ratio of 6–8 for the OA vs. non-OA control at 100 years. Presented data suggest that acquisition of OA substantially contributes to survivability.

### Osteoarthritis tags are most common in long-living subpopulations with significantly delayed cognitive decline

**Figure 4:**
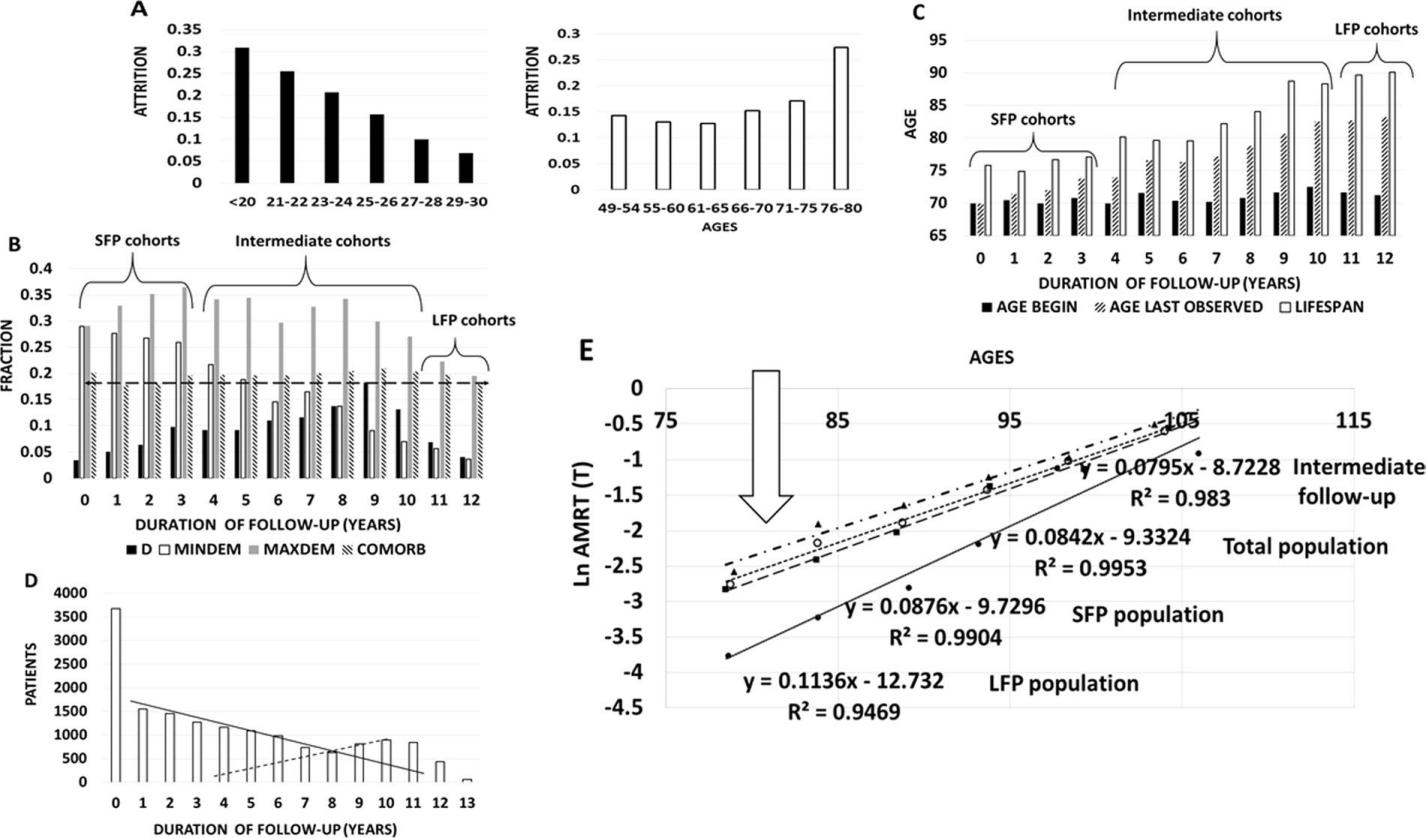
Establishment of follow-up duration as a measure of patient’s dementia resistance and life expectancy by qualitative and quantitative analysis of trends. (**A**) Attrition rate between WAVE1 (2009–2011) and WAVE2 (2012–2013) of TILDA as a function of MMSE score (N = 6300) or age (N = 8512) available in WAVE1 data. (**B**) Distribution of all-cause mortality (black bars), dementia fraction detected at the beginning of follow up on first visit (hollow white bars with a black border), dementia fraction at the end of follow up (grey bars), comorbidities per person at the beginning of follow up and brought to scale by dividing by 10 (striped bars), measured in NACC version 3.0 (N = 15618) as a function of follow-up. The mortality was computed by counting all decedents accumulated during follow up and dividing the number by the initial number in the cohort. Similar method was applied to all-cause dementia, registering all cases that developed by the end of follow up. Long follow-up population (LFP, N = 1700) was compared with short follow-up population (SFP, N = 7300). The dashed line symbolizes similar average number of somatic comorbidities in each cohort at the beginning of follow-up. (**C**) Distribution of achieved lifespans (life expectancy, empty white bars), ages at the start (black bars) and ages at the end of follow up (grey bars) in NACC, version 3.0 (N = 15618) measured in each follow up cohort as a function of follow-up duration. Long follow-up population (LFP, N = 1700) is compared with short follow-up population (SFP, N = 7300). (**D**) Distribution of the numbers of patients in NACC, version 3.0 (N = 15612), identified in each follow-up duration cohort. The trendlines indicate proportion of each hypothetical component (LFP/SFP) in the cohorts formed based on follow up duration. (**E**) Semi-logarithmic plotting of all-cause mortality (AMRT) in the cohorts by age in the NACC sub-populations formed by variable durations of follow up. In the direction of arrow, the regression plots represent: (1) intermediate follow-up duration (N = 5861), (2) total NACC population (N = 14220), (3) SFP sub-population (N = 7078), (4) LFP sub-population (N = 1281). The regression equations are matched to the sub-populations.

Human aging proceeds by varying rates^36,37^. In this report, cohort attrition rates and the lengths of follow up were used as quantitative correlates of aging rates. Figure 4A–D depicts relationships of MMSE scores and attrition between WAVE 1 and 2 of TILDA study (Fig. 4A), between mortality, dementia and follow-up length in NACC study (Fig. 4B), between life expectancy and follow up in NACC study (Fig. 4C) and the age-dependent distribution for the force of failure in the different fractions by follow up in NACC (Fig. 4D).

Figure 4A demonstrates a strong inverse relationship between MMSE score and attrition rate (Pearson’s correlation = −0.979, N = 6300, p < 0.00001 at significance level 0.05). Cohorts stratified by age (as opposed to MMSE stratification) begin to display greater differences in attrition only when cognitive decline becomes pronounced, i.e. in the most senior sub-cohort of 76–80 years old. Of note, in TILDA, increases in somatic comorbidity detectable in sub-cohorts of patients aged 49–54 and 66–70 years fail to reflect on the attrition rate, suggesting that contribution of cognitive decline to attrition is far greater than that for other morbid conditions and even for age up to 75 years cutoff (data not shown).

Figure 4B demonstrates a linear decline in the frequency of dementia diagnosis present at baseline (white bars) in cohorts differing by duration of the follow up, which is an inverse metric to attrition, utilized in NACC, version 3.0, with the Pearson’s correlation −0.989 (p < 0.00001 at N = 15,618 and significance level 0.05). At baseline, dementia was already detected in 29% of patients failing to register for the follow up (FP = 0); on the other hand, only 3.5% of individuals who continued in the study for 12 year were marked with the diagnosis of dementia. The average age at baseline in all the cohorts segregated by duration of the follow up was 70.5 years (Fig. 4C); average amounts of comorbidities in these cohorts were also same (1.9 comorbidities per a person in each cohort), thus, pointing that shorter follow-up and longer follow up cohorts were equivalent in terms of somatic health at baseline. In population analyzed according to follow up lengths, the distribution of mortality rates was somewhat bimodal (Fig. 4B). In sequential cohorts with follow up durations of 1 year to 9 years, the mortality counts AMRT predictably increase, as individuals in longer follow up cohorts achieve advanced age, and cohorts accrue more decedents with passage of more time. However, this trend breaks down in the range of follow-up durations of 10–12 years, when observed mortality decreases despite increasing age of the individuals, in parallel with lower percentage of individuals who demonstrated cognitive decline at baseline.

Same argument applies to all-cause dementia cases accrued by the end of follow up intervals. Amounts of all-cause dementia tags are expected to increase exponentially as a function of follow up length. Instead, these amounts reach a plateau in the follow up range of 3–8 years and start to decline in the range of 9–12 years, despite individuals in this range are substantially older. Figure 4C corroborates the findings of Fig. 4B by demonstrating that life expectancy in the cohorts with follow up range of 9–12 years is approaching 90 years, while life expectancy in the cohorts with follow up of 0–3 years is in range of 75–76 years. Since baseline age of joining the study at 100% survival was 70.5 years in sub-cohorts with various follow up durations, these differences in life expectancy should be attributable to intrinsic differences in aging rates rather than in the difference in age at inception. As the differences in hazard functions attributable to aging process in these cohorts are very substantial, we infer that cohorts with shortest follow-up (0–3 years) and the longest follow up (11–12 years) represent distinct longevity strata co-existing in human population. Figure 4D presents the histogram of NACC Version 3.0 population according to durations of the follow up. The shape of the histogram has bimodal distribution, with the tangent lines delineating the intermediate region where two modes of distribution overlap. Based on these tangent lines, the ranges of 0–3 years and 11–12 years of the follow up were accepted as representing the bulks of the respective subsets intrinsically different in their longevity. Figure 4E formally tests the bi-modality hypothesis by plotting all-cause mortality as a function of age in cohorts with different durations of follow-up. According to this plot, the trends for total population, in cohorts with intermediate follow-up durations and in cohort with 0–3 years duration of a follow-up resemble each other, with the Gompertz parameters being very close to the published ones^36,37^; only 0.8% of entire population exceeds centenarian benchmark. By contrast, the cohort with longest follow-up demonstrates reduced Gompertz alpha constant and increased beta constant, with 11% of individuals reaching 100-year lifespan. These observations indicate that the ability to adhere to a study for a long period of time may serve as a marker for both a resistance to cognitive decline and an overall longevity.

**Figure 5:**
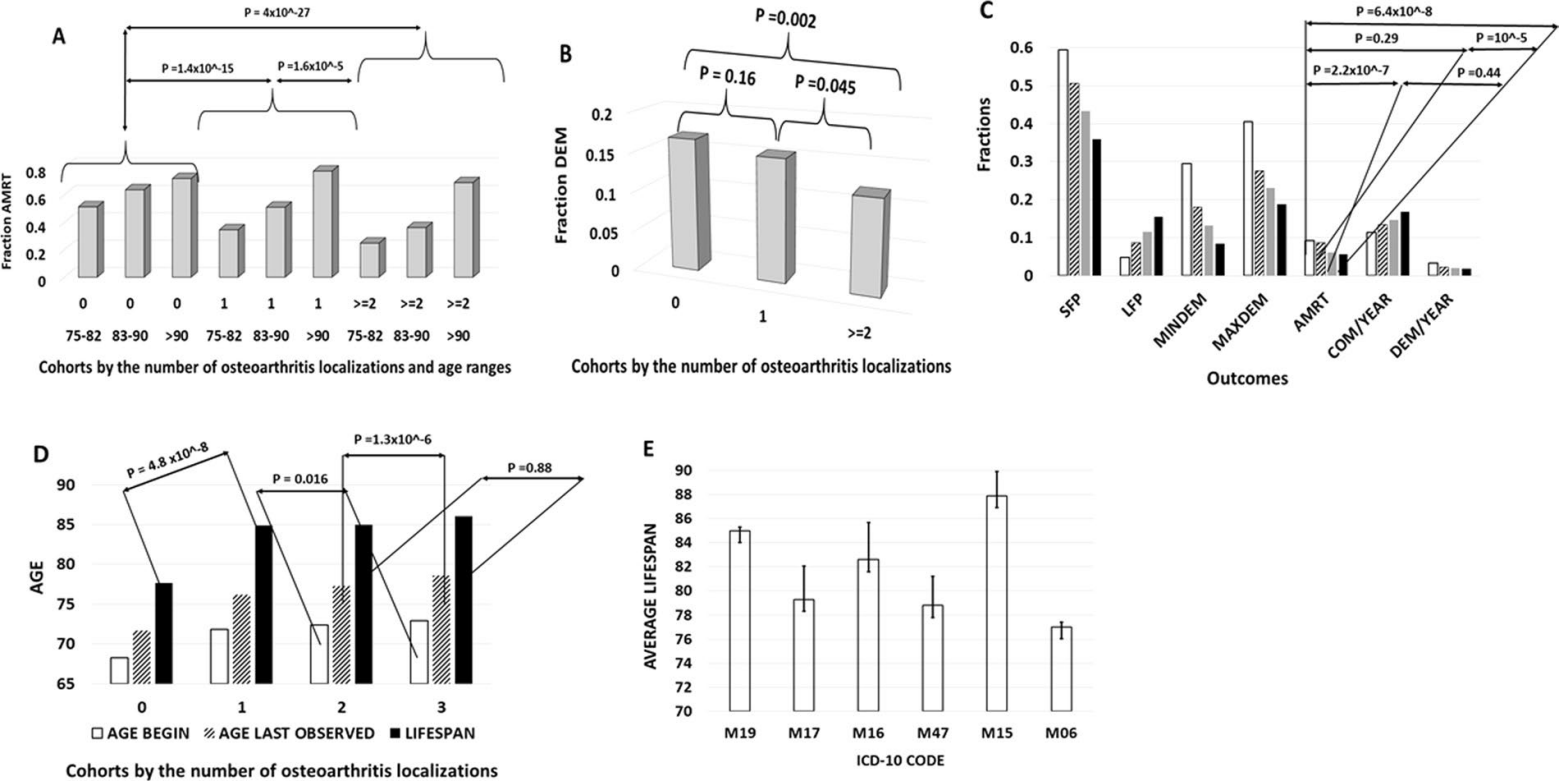
Dependence of the outcomes on the number of osteoarthritis localizations, quantitative analysis of multiple sources. (**A**) Dependence of all-cause mortality (AMRT) on age and the number of osteoarthritis localizations, 5540 patients in Kaiser Permanente sudy of the oldest old. Significance of differences between the cohorts is displayed. N = 3900 for the non-arthritis control (0 localizations), N = 1100 for osteoarthritis in 1 localization, N = 540 for osteoarthritis in 2 and more localizations. The cohorts are balanced by age and gender. (**B**) Dependence of dementia fraction on the number of osteoarthritis localizations, 5540 patients in Kaiser Permanente study of the oldest old. Siginificance of differences is displayed. The size of cohorts is the same as in (**A**). The cohorts are balanced by age and gender. (**C**) Dependence of outcomes in NACC database on the number of osteoarthritis localizations. White bars – control, 0 localizations (N = 6570), striped bars – 1 localization (N = 4015), grey bars – 2 localizations (N = 2830), black bars – 3 or more localizations (N = 2155). The outcomes are SFP – Short Follow-up fraction in the cohort, LFP – Long Follow-up fraction in the cohort, MINDEM – dementia fraction at the beginning of follow up, MAXDEM – dementia fraction at the end of follow up, AMRT – accrued all-cause mortality, COM/YEAR – annual rate of progression of total comorbidity count, DEM/YEAR – annual rate of dementia progression. The statistical significance of differences in AMRT is mapped. The cohorts are balanced by gender composition. (**D**) Dependence of outcomes in NACC database on the number of osteoarthritis localizaitons. The outcomes are the initial age of cohort at the beginning of follow up (white bars), the final age of cohort at the end of follow up (grey bars), the lifespans in the cohort (black bars). Significance of the differences is mapped. 0 – control, 1 – one localization, 2 – two localizations, 3 – three and more localizations. The cohorts are balanced by gender conposition. (**E**) Lifespan in different forms of osteoarthritis in NBER database. M19 – osteoarthritis of hand, N = 4100, M17 – osteoarthritis of knee, N = 62, M16 – osteoarthritis of hip, N = 47, M47 – osteoarthritis of spine, N = 123, M15 – polyosteoarthritis, N = 65, M06* - rheumatoid arthrtiis, N = 3353. The confidence intervals (CI95) are provided. A control with 566036 patients with 1–10 cardiovascular conditions was provided, measuring 76.77 ± 0.02 average lifespan.

Figure 5 describes all-cause mortality, life expectancy, dementia at baseline, enrichment by longest follow-up and accumulation of comorbidities in the cohorts matched by age and gender and differing by extent of osteoarthritic phenotype,

Figure 5A presents mortality trends in Kaiser Permanente study of the oldest old as a function of the number of body sites affected by OA. It was found that sub-cohort with more generalized arthritic process (**≥**2 localizations) is characterized by a more exponential-shaped profile of decreased mortality, with all three cohorts (no arthritis, 1 and **≥**2 body localizations) being statistically distinct. Figure 5B presents a significant decrease in frequency of all-cause dementia in a cohort with OA affecting **≥**2 body sites, as compared to sub-cohorts with no OA and with the disease affecting only one body site.

This trend was reproduced in NACC for multiple outcome metrics (Fig. 5C). In particular, the cohorts with 3 or more body sites affected by OA, which was most depleted in “shortest follow-up” component and most enriched in “longest follow-up” component, had demonstrated the lowest dementia prevalence both at baseline and at the last follow-up as well as the slowest rate of dementia progression. In terms of AMRT, the cohorts with 2 and 3 osteoarthritic body sites statistically differ from that with no arthritis or with one body site affected by OA, while the differences between “OA in 2 body sites” and “OA in 3 body sites” as wells as “no arthritis” and “one body site osteoarthritis” fell short of significance (p = 0.44 for “OA in 2 body sites” vs. “OA in 3 body sites”; p = 0.29 for “no OA” vs “OA in 1 body site”, p = 6.4 × 10^−8^ for “no OA” vs “OA in 3 body sites”, p = 1 × 10^−5^ for “OA in 1 body site” vs “OA in 3 body sites”, p = 2.7 × 10^−7^ for “no OA” vs “OA in 2 body sites”, Fig. 5C).

In NACC, the trends in the rate of the progression for the somatic comorbidities were opposite to the trend for the rate of dementia progression. While somatic comorbidities demonstrated the fastest build-up in the OA cohort with disease affecting at least 3 body sites, the rates of dementia increment in the same cohort were the slowest. Figure 5D follows the same dataset as 5 C, presenting the ages of the cohorts. In the cohort with three or more body sites affected by osteoarthritis, decreased mortality and the prevalence of dementia were accompanied by the highest age at last follow up and the longest lifespan. Age difference between the cohorts of patients with arthritis affecting one or more body sites were relatively small at the baseline, however, these differences increase substantially after follow ups in the respective cohorts (p = 0.016 for the ages of follow up inception between 2 and 3 body site locations, p = 1.3 × 10^−6^ for the ages of the ends of the respective follow ups, p = 4.8 × 10^−8^ for the difference in lifespans between non-OA control and OA in one body site, Fig. 5D). In the NACC v. 3.0 database analysis, statistical differences between the lifespans were impacted by generally low mortality rate, diminishing the tag density (Fig. 4D). The results of lifespan analysis in NBER patients with arthritis affecting various types on body sites are given in Fig. 5E. The ICD-10 code M19 represents OA of hand, with little impact on daily mobility and exercise, explaining relatively higher lifespan (85 years vs. 73 for the general population control and 79 for a cohort labeled with diagnostic tag M17 – arthritis of the knee. Average lifespan of cohort carrying diagnostic tag M15, which represents poly-osteoarthritis, a disease generally associated with diminished mobility, was longest, at 88 years. Thus, analyses of three data sources, Kaiser Permanente, NACC and NBER, show that generalized OA with multiple localizations is associated with more significant reduction of mortality and dementia than a singly localized OA or no arthritis.

## Discussion

In this report we present an observation of a novel biological factor contributing both to longevity and to dementia prevalence in human populations. Specifically, in 7 independent datasets and by meta-analysis of published literature we verified that the diagnosis of osteoarthritis is accompanied by higher cognitive scores at baseline, and correlates with delayed rate of cognitive decline along with decreased all-cause mortality, which are especially prominent in younger cohorts (Figs. 1–3, Tables 1 and 2). In patients with OA, kinetics of aging is altered as compared to non-arthritic controls (Table 3), with the reduced pre-exponential Gompertz constant alpha balancing the increased exponential constant beta in the hazard function of mortality H(T)^36,37^. We identified flexible follow up interval as a metric of patient’s robustness. Specifically, the top ranking 10% ranges of follow up identify a long-living human sub-population with 11% reaching centenarian mark as compared to only 1.1% achieving this longevity in general population^38^. The conclusions made by analyzing U.S. population data were confirmed by analysis of Australian Grim Book^39^ in Table 3, pointing to even greater extension of lifespan in OA cohort observed in longer living population of Australia (82 years vs. 78 years). When individuals were ranked according to the duration of follow-up, the top strata termed LFP (long follow-up population) was found to be enriched with osteoarthritis tags (especially with tag of OA in multiple body sites), forming a convenient marker of longevity and cognitive robustness (Fig. 4). Relying on the markers of cognitive decline and longevity, we identified the number of osteoarthritic body sites as a critical variable (Fig. 5).

Before proceeding to hypotheses attempting biological interpretation of the findings, we should address the challenges of present study.

One challenge is inherent inconsistency of results across datasets, which is evident from data in Table 2. Such inconsistency may result from varying definitions of OA in different datasets (for example, clinician-defined, X-ray defined or self-reported, historical definitions of ICD codes), possible accidental contamination of analytical set with rheumatoid or infection-driven arthritis cases, as well as inherent study biases and the difference of data normalization protocols, cumulatively resulting in obvious spread of the hazard ratios. We approached this inconsistency by meta-analyzing 34 projects led by other teams and 7 large datasets, where OA was clinician-defined, and observed similar trends. Across the sum of evidence presented in this report, we conclude that HR <1.0 is statistically dominant in cohort-weighted averages. As a minimum, existing literature evidence supports our conclusions based on 7 datasets alone, with some references producing HR values approaching the range shown in Figs. 1–3.

Our study explains previously observed inconsistency of the literature describing morbidity and mortality trends in patients with dementia and osteoarthritis. With the trajectory of systemic decline being more exponential in patients with OA (Figs. 1–3, Tables 2 and 3), the populations which are further away from terminal decline in terms of age or health status may experience lower hazard ratios of dementia as compared to non-OA controls, while the age and health strata which are closer to terminal decline experience opposite trend (Fig. 1, Table 2). It seems that the difference in decline trajectories observed in patients with OA and in non-arthritic controls, when combined with longer lifespan observed in OA cohort (Figs. 3C, 4D and 5E), may have some pathophysiological underpinnings deserving detailed exploration.

Another challenge was a gender disparity of OA cohorts and greater polypharmacy scores attained by OA patients, both being possible confounders. Figure 3A presents stratification of NACC data for genders in the context of age-specific mortality in OA cohort vs non-arthritic controls and confirms the trends of reduced mortality in OA for both genders. Multiple regression analysis^40^ shows that the effect of osteoarthritis cannot be reduced to the effect of associated polypharmacy, however, additional investigations of this phenomenon are warranted. Specifically, in NACC and NSHAP datasets where OTC supplement data were available, both OA labels and multiple supplementation labels were behaving as statistically significant and independent factors preventing dementia. Notably, prescription polypharmacy was a factor contributing to the dementia rather than preventing it. Covariation and correlation of the diversity of OTC supplements with overall adherence to a healthy lifestyle, attention to health, and other factors is likely^40^.

Greater multi-morbidity burden and lesser physical mobility commonly observed in OA do not support the hypothesis that OA-associated protection against dementia is provided by arthritis-associated pharmaceuticals and by more frequent doctor’s visits. For example, the data on death certificates collected by NBER, including autopsy reports, support objectively higher multimorbidity burden observed in OA (almost 2-fold above that in control, see M15-M19* ICD-10 codes), with life-threatening disease (cardiovascular, renal, metabolic) being more prevalent in the decedents tagged with OA^38^. In case of NACC, OAI, NBER, GRIM BOOK or KAISER data, all diagnoses were established by clinicians/pathologists and are not self-reported. Decreased all-cause mortality in OA was detected in cohorts with already established diagnoses of other serious diseases such as fibrillation, stroke, infarction, cancer, indicating that the observed differences in mortality cannot be explained by under-diagnosing of serious conditions in non-arthritic controls. In each diagnostic category and especially in a cohort tagged with a combination of diagnoses, OA patients were demonstrating improved survival in strata with same age and gender composition as non-OA controls.

Figures 1, 3 and 5 show 7 to 11-year difference between the lifespans in M05, 06, 17* (short-lived, 76–78 years) and in M15, 19* (long-lived, 85–88 years). With short-lived sub-sets of arthritis patients serving as an internal control and accounting for the biasing factors of additional medication and doctor’s visits, longer-living sub-cohort stands out. Moreover, the most long-living are the poly-arthritic patients with lower body and spinal disks impacted. With decrease in exercise, physical mobility, increased multimorbidity and presence of chronic pain, these patients emerge as the most resilient cohort, in a manner proportional to the number of impacted body sites (Table 3, Figs. 4 and 5). Some previous publications by others have reported similar trends (Table 2), pointing that these striking observations are unlikely to be bias-driven. How an increased attention to one’s health and a daily routine modulated by chronic pain impact the ratio between dementia and the rate of co-morbidity accumulation, differing more than two-fold between OA and control cohorts, remains unclear.

The conclusions presented here are results of observational study, and, therefore, do not provide mechanistic explanations for protection against dementia and for promotion of longevity evident in cohort with osteoarthritis. Based on published literature, one possible hypothesis poses that osteoarthritic tissue may aid in sensitization of immune cells to senescence specific antigens^41^ and, therefore, facilitate immune clearance of cells with Senescence-Associated Secretory Phenotype (SASP)^42,43^. In turn, minimized SASP in microglia may allow for better maintenance of neural stem niches, which is vital for systemic well-being^43^. In fact, macrophages are activated in osteoarthritis^44^, and were identified as the agents responsible for senescent cell clearing^45–48^. Another possible biological explanation for negative association of osteoarthritis with ageing is that OA resembles a “non-healing wound”, which is arrested at inflammatory resolution stage^49^. In OA, but not in RA, body attempts to resolve inflammation by hyperactivation of anti-inflammatory macrophages, evident from an increase in M1/M2 polarization ratio both in synovial fluid and in peripheral blood of OA patients^50–52^. Persistent saturation of peripheral blood with anti-inflammatory signals may also aid in preserving microglia, and anti-inflammatory polarization within this compartment^53^, thus, contributing to OA-attributed delay in cognitive decline.

In conclusion, our meta-analysis of the impact of osteoarthritis (OA) on cognitive decline and overall mortality indicates higher cognitive scores, later dementia onset as well as longer lifespan and lower age-specific all-cause mortality in OA cohorts. Moreover, generalized OA with multiple localizations is associated with more significant reduction of mortality and dementia than a limited OA or no arthritis. It is tempting to speculate that life-extending effects of OA are due to the disease-driven conditioning of immune system.

## Methods

### Data sources

The NBER (National Bureau of Economic Research) database, Multiple Causes of Death^38^ contains scanned data profiles obtained from death certificates, including age, gender, education, demographic and family status of the decedent, number of diseases, ICD-10/358 codes of the individual diseases, and the order in which they are considered. The database includes data from across the USA since 1959 with more than 50 million decedent profiles as of 2016. The coverage for this project was limited to 1999–2016. The ICD-10 code G30.9 was selected to identify Alzheimer’s disease; F02* and F03* were used for other forms of dementia, excluding alcohol-induced; I10-I15*, I20-I25*, I26-I28*, and I30-I52* were selected for cardiovascular disease; M13*, M15-M19*, and M02-M09* were selected for arthropathies; E10-E14* were used for diabetes; C* was used for malignancies.

The GRIM BOOKS database is the multiple cause of death source tracked for Australian population since 1907, with OA data available as of 1978 for M19* code. More than >10^7^ decedents are reported in this source and ICD-10 codes are available^39^.

The NACC database is affiliated with National Alzheimer’s Coordination Center^33^. This source has collected multi-centre data nation-wide since 2007, and the release as of 09/2018 was analysed. The database is organized longitudinally with 138,200 visits for 38,800 de-identified patients, and includes comorbidities, ages of entering and exiting observation, cognitive status (MMSE, MOCA and other), family and demographic data, alive/dead flag and pharmaceuticals taken during each visit. The dosages are not provided by NACC and were assumed to be typical prescription dosages. The clinician-assigned tags for osteoarthritis and rheumatoid arthritis are provided in version 3.0 of NACC data (N = 15692 patients), combined with anatomical localizations.

The National Social Life, Health, and Aging Project (NSHAP) Waves 1 and 2^30^ are affiliated with the Inter-university Consortium for Political and Social Research. The longitudinal dataset (>3000 patients) provides information about supplement and pharmaceutical exposure, comorbidity presence and all-cause mortality. The dementia rate is available but is under-represented compared to the age-matched general population outside of the survey. Cognitive scores (elements of MMSE) were used instead. Osteoarthritis and rheumatoid arthritis tags are available.

The Kaiser Permanente Study of the Oldest Old, 1971–1979 and 1980–1988 [California] is a longitudinal observation study of the elderly >65 years old (>5900 patients) with 35 clinician-diagnosed conditions that were carefully monitored, and dementia data, osteoarthritis, rheumatoid arthritis tags as well as anatomical localizations are available^32^.

The Irish Longitudinal Data on Aging (TILDA) project is a longitudinal study of individuals above 50 years of age in Ireland^31^, which was conducted from 2009 to 2015 in 3 waves. Waves 1–3 were analysed for this project; the averaged Mini Mental State Examination (MMSE) scores were computed, and osteoarthritis, rheumatoid arthritis and MMSE data coexist in the same profiles. Direct data on dementia and mortality are not provided by the publicly released version, but attrition data between the study waves can be produced by linking through patient’s IDs.

The Osteoarthritis Initiative is a multi-center observational nationwide research study directed to prevention and treatment of knee osteoarthritis^35^. The study includes >4900 patients, follow up, OA localization and mortality data.

### Normalization of data

To compare OA and control, the respective cohorts were stratified by age and the mortality rates were compared in the identical age sub-ranges. Genders were considered by stratification first, with each gender being re-stratified by age. Having shown that the relative effects reproduce in both sexes, another approach was to randomly tag excessive male presence in the control and eliminate the excessive male profiles equalizing the gender ratio before age stratification. The tagging was multiply repeated to ensure independence of the result of the exclusion path. Co-morbidities were not included in normalization, since based on the report the biological meaning of comorbidity counts may differ in non-OA and OA systemic contexts. For example, in OA context the patients consume more OTC supplements which can potentially offset the contribution to mortality due to excess morbidity and lesser mobility.

### Definitions, abbreviations and extraction of Gompetz function parameters

Different waves/components of the studies were linked through patient’s IDs. The AGE B (age at the beginning of follow up or baseline age) matches 100% survival and after the period of follow-up (FP) the patients either reach the AGE E (age at the end of follow up) or die and produce lifespan measurement (LS). The interval between LS and AGE B was defined as measured life expectancy (LE). During LE, mortality continues to accrue, producing AMRT (accrued all-cause mortality), an integral metric dependent on the duration of observation and inherent hazard function of mortality (force of failure) H(T)^36,37^. AMRT is measured practically by defining the narrow bracket of ages (5 years) in the category closest to the lifespan (AGE E for the alive component or LS for the decedents) and counting the number of decedents D in the cohort numbering N patients, with AMRT = D/N. The differential Gompert’z function H(T) is defined as a probability of dying within the next year and is written in a simplified form, neglecting non-aging Makeham contribution^36,37^:

1 $${\rm{H}}({\rm{T}})={\rm{\alpha }}\times \exp \,({\rm{\beta }}{\rm{T}})$$

Measured values are produced as:

2 $${\rm{H}}({\rm{T}})={\rm{AMRT}}/{({\rm{LS}}-{\rm{AGE}}{\rm{B}})}_{{\rm{av}}}$$

where (LS − AGE B)_av_ is the cohort-average interval of mortality accumulation to reach AMRT, starting with 100% surviving population at AGE B, α is a pre-exponential Gompertz constant, β is exponential constant, T is the current age. The constants are estimated from semi-logarithmic plotting:

3 $$\mathrm{Ln}\,{\rm{H}}({\rm{T}})=\,\mathrm{ln}\,{\rm{\alpha }}+{\rm{\beta }}\times [{\rm{LS}}]$$

The same methodology was applied to OA (osteoarthritis) and control (CONT) cohorts. Systematic errors arise by postulating a simplified model, due to unequal follow up lengths in different 5-year brackets by age, due to linearization of exponential function in (2) and due to unequal life expectancies in different age brackets. The errors cancel out in the OA/CONT comparisons throughout the report. Internal controls (as opposed to actuarial tables for control) were preferred to ensure error cancelation and agreement with external actuarial data sources was tested by reproducing published data in our computations. The choice of pooling/bracketing method was necessitated by limited volume of available datasets and the need to minimize random variation in each cohort.

### Determining the fraction of population reaching 100-year lifespan

Based on cumulative distribution for Gompertz function neglecting Makeham non-aging component, the time-dependent survival can be described as:

4 $${\rm{S}}({\rm{T}})={\rm{S}}\,({\rm{T}}0)\times \exp \,[-\,({\rm{\alpha }}/{\rm{\beta }})\times (\exp ({\rm{\beta }}({\rm{T}}-{\rm{T}}0))-1)]$$

where S(T) is survival fraction of the population at the age T, S (T0) is the survival fraction at the earlier age T0. Assuming T0 = 0, T = LS = 100, and S(T0) = 1, one obtains a fraction of the population surviving past 100 years benchmark.

### Constructing of empirical prevalence-adjusted survival curve

The method is applicable to MCD (multiple cause of death) sources which do not provide alive component or prevalence of a condition, but merely state the condition as a cause of death in the individual’s record by the respective ICD codes (NBER, GRIM BOOKS). The total number of decedents with the ICD code of interest is grouped by lifespan brackets. In each lifespan bracket the fraction of the total mortality is computed (normalized). The primary age-specific fraction is divided by the fraction of population suffering from this condition at the given age bracket. The age-dependent prevalence information is provided by outside literature sources. The primary fractions adjusted to age-dependent prevalence produce ratios in each bracket which are re-normalized to the total of these ratios, producing prevalence-adjusted fractions. At the age T = 0 the survival S(T) is assumed to be 1.0 and the prevalence-adjusted fractions are subtracted sequentially as a function of age, the residual being the fraction of population surviving after the age T with the given ICD code. This procedure was applied to produce Fig. 3C.

### Meta-analysis of published literature

Published literature concerning mortality rates in OA is presented both in form of individual contributions and meta-analysis reviews. The reviews were excluded. Individual contributions were exhaustively collected by searching both PubMed and Google Scholar with a combination of “osteoarthritis” and “mortality” terms, followed by exploration of semantically similar references by automatic expansion tools. A total of 142 hits were identified in Google Scholar, the references identified in PubMed by the same search technique were same as these retrieved through Google Scholar. The publications that describe ratios of all-cause mortality in OA to that in general population control were collected. Only one publication per each source (research group, first author) was accepted. Publications generated by different research group were assumed to be independent. Selected references were tabulated including volumes of the cohorts and hazard ratios (HR) for mortality rates in different forms of OA. When variable HR within the same publication were reported, the lowest and the highest values in the range were recorded in each individual contribution. Separate integrations of the individual results were reported for the lower and higher levels of HR in each reference. The individual publications were integrated in the meta-analysis using weighting system of Hunter and Schmidt, based on weights defined as the number of patients in a cohort^54^.

### Determining the choice of a reported outcome at a fixed cohort size

With the cohorts of the fixed size, rare tag occurrence may produce random fluctuations exceeding the size of the intended measured effect. The tags were screened for the adequate prevalence in the cohort to report the effects with maximized signal-to-noise ratio.

5 $$[{\rm{Cohort}}\,{\rm{size}}] > {\rm{NC}}={{\rm{Z}}}^{2}\times ({\sigma }^{2}/{{\rm{e}}}^{2})\times ({\rm{M}}/[{\rm{Cohort}}\,{\rm{size}}])$$

where NC – the requested sample size, Z – z-score of the confidence probability equal 1.98 for α < 0.05, e – acceptable value of noise-to-signal ration, assumed 0.1, σ - variation of the tag prevalence between random groups of the size M, M/[Cohort size] – adjustment of the variation determined in a one-time calibration for the random group of size M to various sizes of the cohorts, see^55^. The values of NC were computed for different possible outcomes with different relative variation σ and those that satisfied (5) were acceptable.

### Assumptions in assessments of statistical significance and correlative relationships

Significance of observed differences was measured by 2-sided heteroscedastic T-test without any assumptions about equality of variances in the samples or relative positioning of the means. Confidence intervals were measured as CI95 (95% probability of the true value situated within the computed interval) unless specified otherwise. Correlation coefficients between the profiles of numbers of variable length were assessed for reliability, with p-value <0.05 of null hypothesis (actual non-correlation imitated by random arrangement) being the cutoff.

## Supplementary information

Meta-analysis of published literature

Table of supplemental files and datasets

## Data Availability

All data generated or analyzed during this study are included in this published article (and its Supplementary Information Files).
