# Supplementary material for: Increased lifespan, decreased mortality, and delayed cognitive decline in osteoarthritis
Source: Sci Rep. 2019 Dec 9;9:18639. doi: 10.1038/s41598-019-54867-8 (PMC6901554; doi:10.1038/s41598-019-54867-8)
Supplement: Supplementary file 2 — Table of supplemental files and datasets [file 41598_2019_54867_MOESM2_ESM.docx]

**Supplemental files for the article “Increased lifespan, decreased mortality, and delayed cognitive decline in osteoarthritis”.**

**By Dr. Anatoly Mayburd and Prof. Ancha Baranova**

| **File name** | **Link** | **Description** |
| --- | --- | --- |
| **TILDA1 Codebook.pdf** | **https://osf.io/zr4v5/** | **Codebook for TILDA 1 wave of the survey** |
| **TILDA2 Codebook.pdf** | **https://osf.io/csekj/** | **Codebook for TILDA 2**  **wave of the survey** |
| **GRIM BOOKS ORIGINAL DATA - Table 3 contribution.csv** | **https://osf.io/eyz38/** | **Original data downloaded from the Australian multiple cause of death source titled “GRIM BOOKS”** |
| **KAISER PERMANENTE-Codebook.pdf** | **https://osf.io/xs8wg/** | **Codebook for the KAISER PERMANENTE STUDY OF THE OLDEST OLD.** |
| **Kaiser Permanente - Figures 1C-D, 5A, 5B.xlsx** | **https://osf.io/pjd3k/** | **Kaiser Permanente data supporting the figures 1C-D, 5A, and 5B of the manuscript.** |
| **NACC 2018 - Figures 2A-C, 4C-E, 5C-D, Table 3 contribution.xlsx** | **https://osf.io/rkvgd/** | **NACC data, release as of 09/2018, supports the Figures 2A-C, 4C-E, 5C-D, Table 3. Only version 3.0 of NACC dataset was accepted.** |
| **NACC 2018 Original data, September freeze.xlsx** | **https://osf.io/z2t76/** | **NACC 2018 Original data, the entire dataset.** |
| **NACC Codebook.pdf** | **https://osf.io/d2e47/** | **NACC Codebook** |
| **National OAI project - Figure 3B.xlsx** | **https://osf.io/34zvx/** | **National OAI projects – support for Figure 3B.** |
| **National OAI project** | **https://osf.io/54vx2/** | **National OAI project** |
| **TILDA - Figure 4A-B.xlsx** | **https://osf.io/xwbyd/** | **Support of Figures 4A-B, based on TILDA** |
| **TILDA - Figure 1B, Table 1, Table 3 contribution.xlsx** | **https://osf.io/9tzsr/** | **Support of Figure 1B, Table 1, Table 3 based on TILDA** |
| **NSHAP WAVE1 2005-2006 Code Book.pdf** | **https://osf.io/536n2/** | **WAVE1 2005-2006 Codebook for NSHAP** |
| **NSHAP WAVE 2 2010-2011 Code Book.pdf** | **https://osf.io/rdsfz/** | **WAVE2 2010-2011 Codebook for NSHAP** |
| **NSHAP - Figure 1A.xlsx** | **https://osf.io/2e6ym/** | **NSHAP – supporting data for Figure 1A.xlsx** |
| **NBER - 2016 data - Figure 3C.xlsx** | **https://osf.io/uqnck/** | **NBER – 2016 data – Figure 3C support** |
| **NBER - 2011 data - Figure 5E.xlsx** | [**https://osf.io/9tgj6/**](https://osf.io/9tgj6/) | **NBER – 2011 data – Figure 5E support** |
